# Estimation of the Botanical Composition of Clover-Grass Leys from RGB Images Using Data Simulation and Fully Convolutional Neural Networks

**DOI:** 10.3390/s17122930

**Published:** 2017-12-17

**Authors:** Søren Skovsen, Mads Dyrmann, Anders Krogh Mortensen, Kim Arild Steen, Ole Green, Jørgen Eriksen, René Gislum, Rasmus Nyholm Jørgensen, Henrik Karstoft

**Affiliations:** 1 Department of Engineering, Aarhus University, Finlandsgade 22, 8200 Aarhus N, Denmark; madsdyrmann@eng.au.dk (M.D.); rnj@eng.au.dk (R.N.J.); hka@eng.au.dk (H.K.); 2 Department of Agroecology, Aarhus University, Forsøgsvej 1, 4200 Slagelse, Denmark; anmo@agro.au.dk (A.K.M.); rg@agro.au.dk (R.G.); 3 Agro Intelligence ApS, Agro Food Park 13, 8200 Aarhus N, Denmark; kas@agrointelli.com (K.A.S.); olg@agrointelli.com (O.G.); 4 Department of Agroecology, Aarhus University, Blichers Allé 20, 8830 Tjele, Denmark; jorgen.eriksen@agro.au.dk

**Keywords:** deep learning, clover-grass, precision agriculture, dry matter composition, proximity sensing

## Abstract

Optimal fertilization of clover-grass fields relies on knowledge of the clover and grass fractions. This study shows how knowledge can be obtained by analyzing images collected in fields automatically. A fully convolutional neural network was trained to create a pixel-wise classification of clover, grass, and weeds in red, green, and blue (RGB) images of clover-grass mixtures. The estimated clover fractions of the dry matter from the images were found to be highly correlated with the real clover fractions of the dry matter, making this a cheap and non-destructive way of monitoring clover-grass fields. The network was trained solely on simulated top-down images of clover-grass fields. This enables the network to distinguish clover, grass, and weed pixels in real images. The use of simulated images for training reduces the manual labor to a few hours, as compared to more than 3000 h when all the real images are annotated for training. The network was tested on images with varied clover/grass ratios and achieved an overall pixel classification accuracy of 83.4%, while estimating the dry matter clover fraction with a standard deviation of 7.8%.

## 1. Introduction

Estimation of the clover/grass ratio is essential for optimal fertilization, as it has significant economic potential for the dairy industry. Clover-grass combines high productivity and low environmental impact if the nitrogen (N) supply is adjusted according to the actual clover content. Nonetheless, manure and fertilizers are used largely irrespective of clover content due to poor ability to assess clover content and lack of knowledge on the correlation between fertilizer (N) and clover content during the season.

A high clover fraction gives a higher feed uptake and thus higher performance for cows when the harvested material is used for fodder. The goal is, therefore, to get a larger clover percentage into the clover pastures. Clover is an N-fixing crop able to capture and utilize free N from the air, while grass relies on soil available N for its growth and development. Clover is, however, also able to take up and utilize N from the soil, which creates competition between grass and clover if there is a sufficient amount of soil N available. In cases of low soil-available N the clover will outcompete the grass crop. However, due to its faster growth, the grass will outcompete the clover crop if high amounts of soil N are available. Therefore, it is possible to control the competition and thereby the clover-grass ratio by applying more or less N. Determining the clover-grass ratio thus automatically has great potential for optimizing N applications.

Clover and grass are often grown in mixtures, as clover-grass leys with different species increase the yield stability [1,2] and herbage quality [3] compared to fertilized grass-only leys. This is due to niche complementarity [4] and the greater protein content of the clover [5,6]. Thus, properly managed clover-grass mixtures produce a greater yield compared to pure stands of grass and clover [7,8]. In order to implement targeted fertilization in practice, it is important to firstly estimate actual clover content in a sward and, secondly optimize fertilization based on clover content.

In agriculture today, the amount of N added is based on coarse visual inspections, which are subject to errors and do not cover the variability within a single field or between fields. Automated and precise methods for estimating clover/grass ratio could potentially improve the fertilization strategy, thus improving yield and quality of biomass, resulting in a great financial impact. Positive environmental impacts include reduced N leaching (as more N will be absorbed and utilized by the clover-grass), and potentially reduced CO_2_ emissions, as some fertilization operations can be skipped if the correct amount of clover is present early in the life-span of the clover-grass field [9,10].

Researchers have previously sought to estimate the content of clover-grass in images. Bonesmo et al. [11] developed an image processing software program for pixel-wise classification of clover-grass images. The program used color indexes, edge detection, and morphological operations on the color images to distinguish and quantify the amount of soil, grass, clover, and large weeds. The software estimations and the manually-labeled clover coverage showed a high correlation (r^2^ = 0.81).

Himstedt et al. [12] used digital image analysis on images of grass-legume mixtures from a pot experiment to determine the relative legume dry matter contribution. The image analysis was used to determine the legume coverage (red clover, white clover, or alfalfa) by applying a sequence of morphological erosions and dilations. In each image, the legumes were manually encircled to determine the actual legume coverage. The estimated legume coverage showed a high correlation to both the actual legume coverage (r^2^ = 0.79−0.87) and the relative legume dry matter contribution (r^2^ = 0.82). Himstedt et al. [13] proposed an extension to estimate the absolute legume contribution using legume coverage, total biomass, and logit transformation. Himstedt et al. [14] improved on the image analysis method by filtering the images in hue, saturation, and lightness (HSL) space and applying color segmentation to separate plants and soil after a sequence of morphological operations.

Rayburn [15] correlated randomly sampled points in clover-grass images to relative legume and grass fractions. A regularly spaced grid was placed on top of each image at a random location, and the pixel at each grid location was manually classified. The distribution of classes was used to estimate the relative fractions. The estimated grass and legume fractions were not significantly different from the measured fractions and they showed a high correlation (r^2^ = 0.96−0.98).

McRoberts et al. [16] used local binary patterns to estimate the grass fraction from color images converted to grayscale. The images were tiled into 64×64 pixel blocks, which were manually labeled as either legumes, grass, or unknown. The local binary patterns were made to be rotational invariant by grouping rotational similar patterns together. A histogram of the local binary pattern groups along with the height were used to estimate the grass fraction with a strong correlation (r^2^ = 0.81−0.85).

Automatic segmentation of images from clover-grass fields is a research area with great potential in precision agriculture. Nevertheless, there has been limited success in automated estimation of clover and grass content from red, green, and blue (RGB) images so far. The primary limitation of current methods involve the uncertainty in the image recognition process. Although the use of morphological operations has been shown to correlate with the clover content in the images, it lacks robustness with regards to scale invariance, field conditions, and estimation uncertainty. With varying clover sizes, camera or vegetation heights, or camera resolutions, the parameters of the existing methods need adjustments. This is illustrated by the drop in performance in the work of Himstedt et al. [13] when moving from green house pots to field conditions.

**Figure 1:**
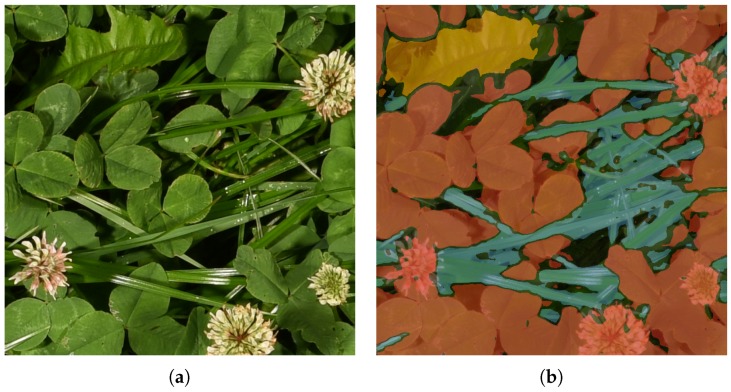
Example of the image analysis on a real image. Each pixel in the image is automatically analyzed and classified as either grass (blue), clover (red), weeds (yellow), or unidentified (black overlay). (**a**) Example input image; (**b**) Automatically analyzed image.

The aim of this project is to automatically determine the clover fraction in clover-grass fields through the use of machine learning to analyze RGB images for grass, clover, and weeds. We follow the procedure of splitting the challenge of estimating the clover content in dry matter from images into a two-step problem, similarly to Himstedt et al. [12]. However, instead of relying on simple patch detections using morphological operations, we propose training a deep convolutional neural network, based on the fully convolutional network (FCN) architecture [17], to directly classify relevant plant species visible in the images [18,19]. This provides the latter stage of clover content estimation with information on the field patch composition and the detected coverage of grasses, clovers, and weeds in the image. Specifically, the selected convolutional neural network is designed to output a semantically segmented image, specifying the plant species of every pixel in the image. An example of such automatic semantic segmentation is shown in Figure 1, where pixels classified as grasses, clovers, and weeds are visualized by blue, red, and yellow overlay, respectively.

The main challenge of moving from manually designed features of the previous methods to learned features using neural networks is the significant increase in demand for ground truth data. To obviate this demand, a simulation environment was designed to generate labeled images of specific field compositions, from which the discriminating features are learned. The trained neural networks were then evaluated against human-annotated images for pixel-wise classification. Finally, a trained network was combined with the latter processing stage to estimate the clover content of the dry matter directly from images.

## 2. Materials and Methods

### 2.1. Data Material

In order to evaluate the image segmentation, clover-grass field trial samples were photographed, harvested, and separated into distinct fractions of ryegrass, white clover, red clover, and weeds. The images and corresponding biomass samples represent the physical data of this paper. There are two uses for these samples:

1. Evaluation of the use of convolutional neural networks (CNNs) for semantic segmentation on a hand-annotated subset of real images.
2. Application of the CNN on the gathered images and validation of the coupling between semantic segmentation of the clover grass canopy and the dry matter species composition in the yield.

#### 2.1.1. Field and Agricultural Setup

The primary samples were collected in 2017 within a primary experimental site measuring 2 hectares at Aarhus University, Foulum (world geodetic system 1984 (WGS84): Latitude 56.495365, Longitude 9.569537). The field trial consisted of 60 clover-grass plots, ranging from 1 to 4 years in age (established in 2013–2016). A mixture containing 82% perennial ryegrass (*Lolium perenne* L.), 14% white clover (*Trifolium repens* L.), and 4% red clover (*Trifolium pratense* L.) with respect to the seed weight was seeded. To increase the variations of the clover-grass ratios for this research, five different fertilization strategies (0, 50, 100, 200, and 300 kg N/hectare) were applied in a randomized design. All fertilization was applied as cattle slurry injected into the soil and no pesticides were used. The site follows a four cut per year strategy, typical for Danish conditions.

#### 2.1.2. Clover-Grass Sample Pairs

Each clover-grass sample gathered in the plots consisted of an image of a defined patch of 0.5×0.5 m and a botanical dry matter composition of the harvested plants in the same patch. These cross-domain samples of both images and dry matter composition are from this point on referred to as sample pairs.

At the time of this research, three of the four seasonal cuts of the 2017 season were performed and analyzed. Acquisition of one sample pair per plot per cut led to 179 acquired samples (one sample was lost due to human error). The sample pairs were gathered on 29 to 31 May, on 6 July and on 10 August 2017. Subsequent to the sample pair acquisition, the whole trial field was cut evenly.

The procedure for sample acquisition was as follows:

1. Capture of an image of the clover-grass without interfering with the vegetation.
2. Placement of a 0.5×0.5 m frame onto the vegetation.
3. Capture of an image to record the placement of the frame and cropping of the initial image accordingly.
4. Harvesting of all vegetation with stems inside the frame at a height of 5 cm.
5. Separation of all plants as either ryegrass, white clover, red clover, or weeds.
6. Drying of the fractions at 60 °C for 14 h.
7. Weighing of the dry matter content of each fraction.

Due to the procedure of harvesting the plants based on stem placement, a potential mismatch was introduced between the photographed canopy and the analyzed dry matter. In homogeneous swards this error will approximately even out, due to the similarity of plants being removed and added to the cut. In sparse or inhomogeneous clover-grass swards, however, this uncertainty adds unwanted noise to the gathered sample pairs, especially in the presence of plants with high coverage such as red clover, dandelions, and thistles.

**Figure 2:**
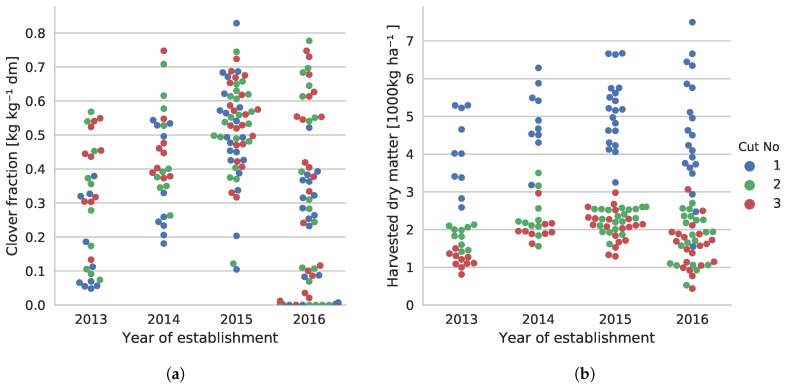
Overview of the variation in the 179 sample pairs sorted by the age of the swards. (**a**) Clover content in harvested material; (**b**) The total dry matter yield of harvested samples. The year axis is discrete, but points are spread out to avoid occlusion of samples.

Figure 2 shows the variations in the collected sample pairs in terms of clover content in the dry matter composition and the total yield. The very low clover fraction in multiple samples of the 2016 establishment is due to poor plot establishment.

#### 2.1.3. Camera Setup

**Figure 3:**
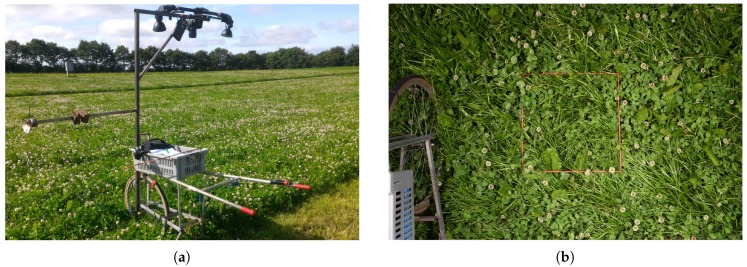

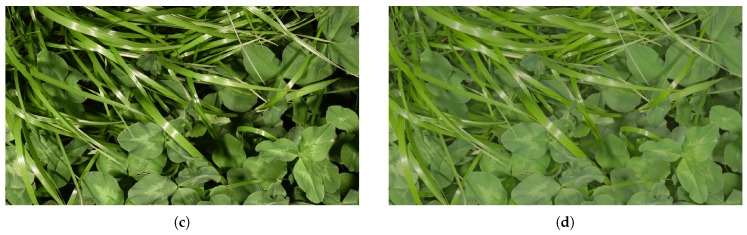
Depiction of the camera setup used for the sample acquisition and visualization of the gamma correction used to reduce the impact of shadow regions. (**a**) The pushcart with the camera system mounted on top; (**b**) Sample of acquired image; (**c**) Crop of an original captured image to illustrate the level of details; (**d**) Gamma-corrected version of (**c**) with a more uniform lighting and increased visibility.

The core of the camera setup consisted of a Nikon D810A camera with two external light-emitting diode (LED) flashes. This setup was mounted on a push cart to fix the capture height, minimize movement during shooting, and ease the transportation in the field. The complete camera platform is shown in Figure 3 along with a sample image. The full 14-bit image resolution was exploited to reduce shadow regions in the vegetation using gamma correction and to correct for uneven lighting of the flashes as demonstrated in Figure 3c,d. Depending on the vegetation height and camera position, the gathered images had a ground sample distance (GSD) of 4–6 pixels per mm.

#### 2.1.4. Image Labeling

For evaluation of the semantic segmentation performance, a subset consisting of 10 of the sample pairs was selected for hand labeling. The subset was designed to cover the variation in the clover content by selecting the image sample closest to each of the the following clover dry matter contents: 5,10,20,30,40,50,60,70,80, and 100%. However, a sample with 100% clover content did not occur naturally in the field plots. Therefore, a single image sample from another dataset was included to maximize the range for semantic segmentation evaluation.

**Figure 4:**
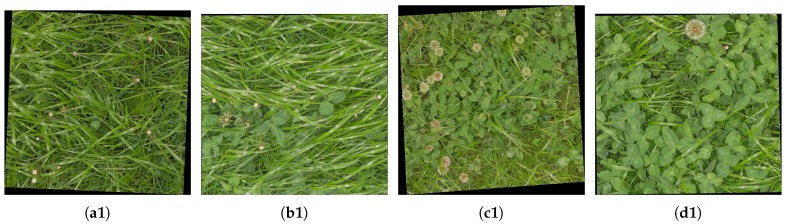

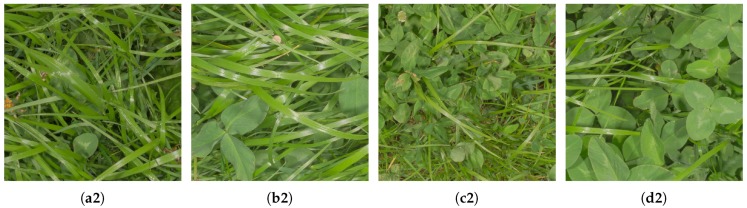
Visualization of annotated image samples. Top row shows four samples with different clover contents. Bottom row shows crops of the images from the top row to illustrate the image resolution. The black pixels surrounding the image samples mark the border of the harvested samples. When the orientation of the camera does not align perfectly with the harvested patch, the image sample appears to be rotated. (**a1**) 5% clover; (**b1**) 20% clover; (**c1**) 50% clover; (**d1**) 70% clover; (**a2**) Annotated image patch of (a1); (**b2**) Annotated image patch of (b1); (**c2**) Annotated image patch of (c1); (**d2**) Annotated image patch of (d1).

To reduce the time requirement for the hand-annotation task, a center crop of 1000×1000 pixels marks the area of the final annotation for each image in the subset. Four samples of the subset are shown in Figure 4 in the form of the original framed clover-grass image samples and the corresponding crops.

#### 2.1.5. Validation Field

To validate the proposed method on a broader basis, additional sample pairs were gathered from field trials at the DLF Seed & Science clover-grass breeding facility in Stevns, Denmark (WGS84: Latitude 55.33916, Longitude 12.38562). The sample acquisition procedure and equipment follow what was described in Section 2.1.2 and Section 2.1.3. The field trials were established in 2015 and 2016 and consisted of ryegrass and white clover mixtures of numerous cultivars. In total, 50 plots were selected by visual inspection to maximize the spread in clover fraction, total yield, time since establishment, stress levels, and phenotypes. The resulting 50 validation sample pairs were acquired on 20 June 2017, as part of the second cut of the season.

### 2.2. Image Simulation

Convolutional neural networks require hundreds, or even thousands of labeled images for training [20]. In the case of semantic segmentation, where each pixel needs to be labeled, achieving this amount of data manually is unfeasible within a reasonable amount of time. This is particularly so in images of species mixtures, where leaves from different species occlude and intertwine with each other and thereby increase the complexity of the labeling task. As an example, it took an average of 3.5 h to annotate each of the 10 images (described in Section 2.1.4), all of which were of 1 megapixel. If all 179 primary samples were to be annotated for training, this would correspond to more than 3000 h of labor.

**Figure 5:**
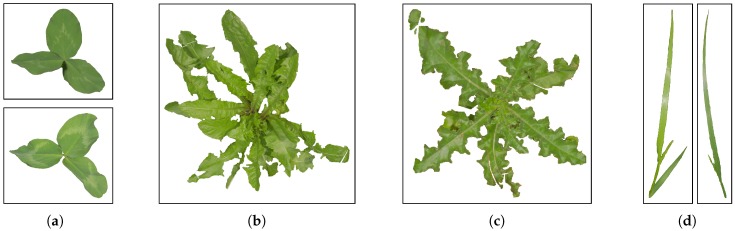
Samples of single plants that are used for simulating images. (**a**) clover; (**b**) dandelion; (**c**) thistle; and (**d**) grasses.

**Figure 6:**
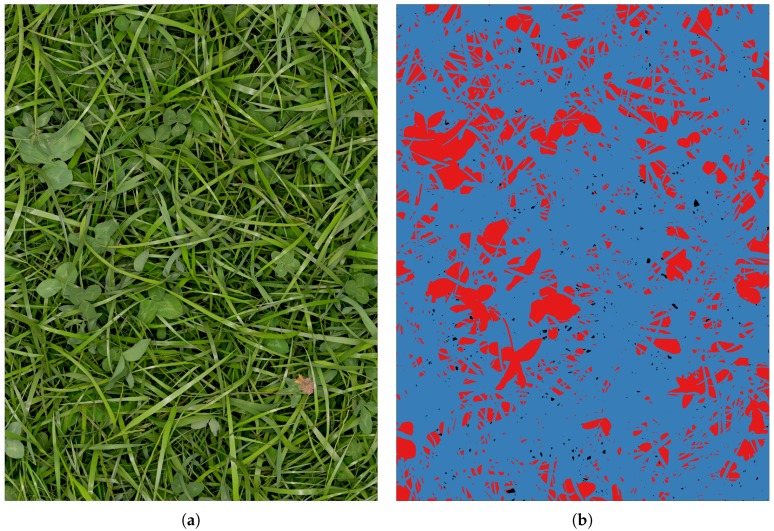
Simulated training data pair. (**a**) Simulated red, green, and blue (RGB) image; (**b**) Corresponding label image. Grass, clover and soil pixels in the RGB image are denoted by blue, red and black pixels in the label image, respectively.

Dyrmann et al. [18] showed how a convolutional neural network can be trained to segment weeds and maize from each other when using only simulated images for training. Inspired by this work, a program which simulates images from clover-grass fields was created. The images are simulated using few hand-segmented samples of clovers, grasses, and weeds, which are placed on top of an image of soil. All the plant samples were acquired from clover-grass field images and selected samples are illustrated in Figure 5. The weeds include dandelion, thistle, and shepherd’s purse. While generating simulated images, corresponding label-images are automatically generated. A label can take one of the following four classes: *clover*, *grass*, *soil*, and *weed*. A sample of a simulated images and its corresponding label is shown in Figure 6.

In total, 55 samples of grass, 117 samples of clover, 14 samples of dandelion, 5 samples of thistle, and 4 samples of shepherd’s purse were used for simulating the images. The total number of plant samples was 195. The effect of varying the number of plant samples is discussed in Section 3.1.

To reduce biases in the species distributions and introduce high levels of variability in the simulated images, several field compositions were simulated. Throughout the field compositions, the clover-grass plant ratio was uniformly distributed. Three different ratios of weeds were used, ranging from none to one-eighth of all the plants in the image. The simulated field density was varied from approximately 10 plants per square meter to complete coverage of the soil. Finally, the clovers were simulated with and without flowers to imitate differences between seasonal cuts. In total these variations amounted to 46 distinct field compositions with individual minor perturbations per simulated image. To train the neural network, 2810 simulated clover-grass images and corresponding label images were generated.

To increase the use of the plant samples and extend the variability of the simulated images, each plant sample was randomly augmented with respect to rotation, scale, and saturation before being placed in the simulated image. The angle of rotation was sampled from a discrete uniform distribution $U\left(0^{\circ },359^{\circ }\right)$ with $1^{\circ }$ increments, the scale was sampled from a uniform distribution in the interval ±25% of the original size, and the saturation was sampled from a uniform distribution in the interval ±25% of the original saturation. Furthermore, the depth was simulated by applying Gaussian shadows to plants when placing them in the image. Applying the large Gaussian kernel to a binary mask of the plant caused thin grasses to cast less shadow than larger clover leaves, leading to a satisfying visual effect. The shadows were added iteratively together with each plant instance, causing underlying soil and plants to become slowly darker. This effect is clearly seen in Figure 6a where areas with lower-placed vegetation appear darker.

### 2.3. Convolutional Neural Network

**Figure 7:**
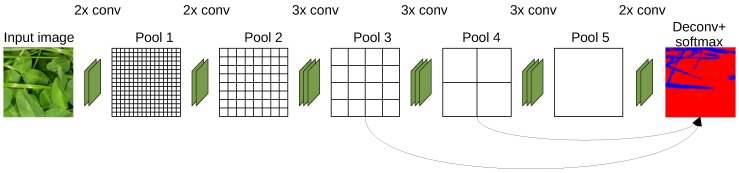
The fully convolutional network with a output stride of 8 (FCN-8s) architecture. The network consists of 15 convolutional layers and 5 max pooling layers. The outputs from pooling layers three and four were routed through deconvolution layers to help restore smaller details in the segmented images. (This figure is redrawn from Long et al. [17]).

Since the introduction of AlexNet by Krizhevsky et al. [21] in 2012, convolutional neural networks (CNNs) have excelled in image classification tasks. These tasks are accomplished by learning abstract features using stacked layers of convolutions and non-linearities by backpropagating thousands of labeled training images through the network and updating the filter kernels accordingly. Contrary to image classification CNNs, the semantic segmentation task of this paper requires a classification output at each spatial position of the input image. This is traditionally accomplished by replacing fully connected layers with convolutions, to maintain spatial feature maps throughout the network, leading to a fully convolutional neural network. In this study, the fully convolutional network with a output stride of 8 (FCN-8s) architecture by Long et al. [17] was used. This model is a modification of the VGG16 architecture [22], which was made fully convolutional by transforming the fully connected layers to convolutional layers, while preserving the learned parameters. The network contains 15 convolutional layers, inbetween which there are five 2 × 2 max-pooling layers with a stride of 2. Each of these pooling layers downscales the input by a factor of 2 horizontally and vertically. After the fifth pooling layer, the image is therefore downscaled by a factor of 32. The pooling layers enable the network to learn semantics, at the expense of fine details, which are scarified. The last convolutional layer and two intermediate layers are followed by deconvolutional layers that up-sample the network output to the size of the input image. In order to compensate for the loss of details and spatial information, shortcuts are made from pooling layers three and four to the deconvolutional layers, whereby finer details can be partly restored. The network architecture is sketched in Figure 7.

#### Training

The CNNs trained for this research followed the same procedure with regards to data augmentation and hyperparameters as the original architecture and were exclusively trained on simulated clover-grass images and labels. The networks’ weights were initiated based on a pretrained VGG16 for image classification [22], and later trained for 12 epochs with a learning rate of $10^{-3}$, weight decay of $10^{-4}$ and mini-batches of four image crops of 1000×1000 pixels for semantic segmentation. The resolution of the original simulated images is comparable to the gathered clover-grass images of 4–6 pixels per mm. To increase the robustness of the network towards images captured with various resolutions, at different heights and different plant sizes, the simulated images and corresponding label-images were augmented by scaling the images prior to training. The images were scaled to 100%, 75%, 50%, and 25% of their original size, which effectively increased the training data fourfold.

## 3. Results

As a result of splitting the task of determining the clover fraction into two problems, the presented results were separated similarly. First, the ability to semantically segment 10 test images was evaluated, followed by evaluation of the dry matter contents on all the gathered sample pairs.

### 3.1. Image Segmentation

To investigate the requirements of using real plant samples for clover-grass simulations, as shown in Section 2.2, the same network architecture was trained twice with different numbers of training samples. Therefore, the two trained models only differed in terms of the plant variation in the training images.

One model was trained on simulated images from all 195 plant samples. The other model was trained on simulated images from a subset of the 195 plant samples. The subset of plant samples was selected randomly and made up 25% of the 195 plant samples.

The FCN-8s models, trained on simulated data, were evaluated with respect to the quality of the semantic segmentation of real images into clover, grass, and weed pixels. This was tested on the 10 hand-annotated image crops. Following the accuracy metrics of Long et al. [17], the pixel-wise accuracy, mean intersection over union, and frequency-weighted intersection over union were calculated for each model and are shown in Table 1.

For a fair comparison to the state of the art, the morphological approach of Bonesmo et al. [11] was implemented and evaluated. The test images for this approach were downscaled to match the original GSD stated in the work by Bonesmo et al. [11]. Additionally, the parameters were fine-tuned to the images, since the provided parameters led to poor classification results.

The FCN-8s architecture has a receptive field of 404×404 pixels, defining the area of the input image used for each pixel classification. Along the borders of the image this context information is reduced, leading to a reduced accuracy in the border region. This effect is particularly pronounced when the image size is reduced, as the border region takes up a larger portion of the total image area. To avoid this effect, the test images were semantically segmented in their original sizes, and later cropped, leading to a useful context in the evaluation.

From the segmentation results shown in Table 1, the improvement from the previous method is clear. The trained convolutional neural network increased the pixelwise accuracy by 17.9 percentage points due to the much higher level of abstraction.

**Figure 8:**
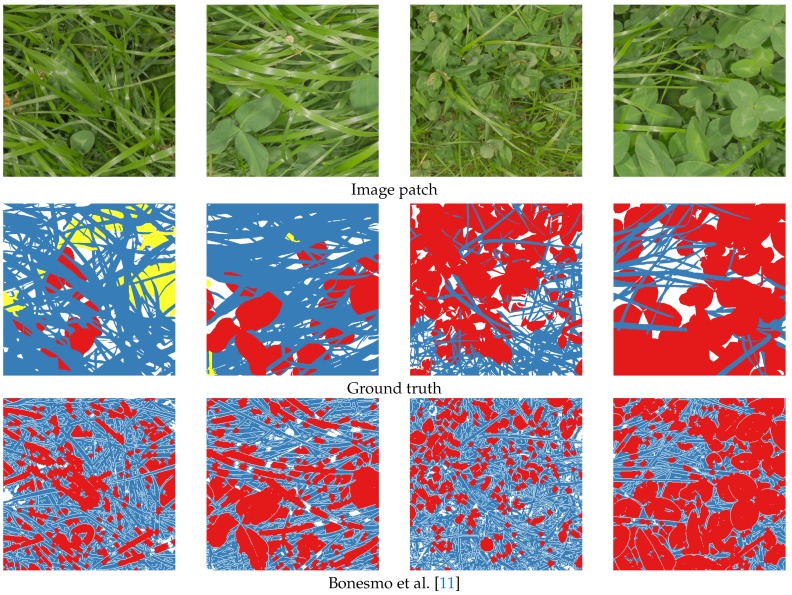

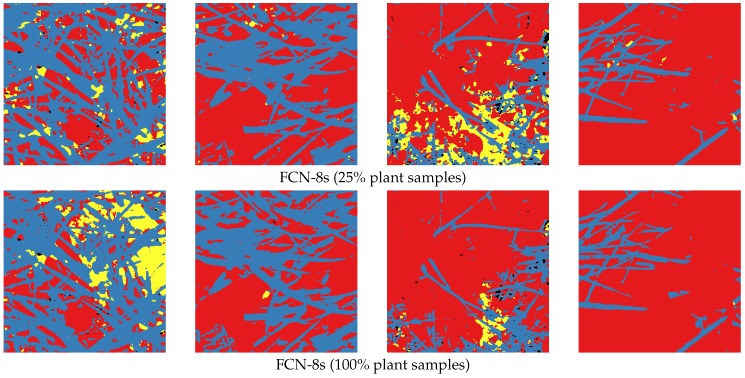
Comparison between four ground truth labels of the hand-annotated images, our implementation of the approach by Bonesmo et al. [11], and the two instances of convolutional neural network (CNN)-driven segmentation. The areas in blue represent grass, those in red represent clover, those in yellow represent weeds, those in black represent soil, and those in white represent areas that could not be distinguished in the manual annotation.

Qualitative results of the semantic segmentation with the varying clover, grass, and weed densities of Figure 4 are shown in Figure 8. Several observations regarding the FCN-8s models are clearly visible in the four samples:

1. The networks learned to identify both grass and clover despite heavy occlusion.
2. Use of 100% of the samples resulted in a great improvement in weed detection. The majority of the weeds were correctly identified in the first column, while the number of false positives in the third column was reduced.
3. Both FCN-8s models were slightly biased towards detecting clover. While the classified grass and weed pixels appeared to follow the contours of the plants in the image, the clover classification appeared to be the default choice of the network. This was beneficial in the fourth row, but led to general misclassifications in the second and third rows.

These observations are supported by the quantitative results in Table 1. The networks reached a high pixel-wise accuracy of 82.2% and 83.4% for 25% and 100% samples, respectively. The increase in mean intersection over union (IoU) was of 3.2 percentage points when moving from 25% to 100% samples, while the frequency-weighted increase was of 1%, supporting the argument that the less-present class of weeds is classified much better.

The approach of Bonesmo et al. [11] can in some cases detect clovers and grasses with high accuracy. This was clearly demonstrated in the fourth column. The morphological operations were, however, challenged by the varying leaf sizes across the test images. This was exemplified in the second and third column, where wide grass was classified as clover, and small clovers were classified as grass, respectively.

The possible use of leaf texture and the surrounding context by the FCN-8s network is believed to be of high importance when segmenting clover grass mixtures. Due to the high level of occlusions in the images, only a fraction of every plant is typically visible, while the leaf texture of the visible part remains.

### 3.2. Clover Fraction Estimation

To verify the usefulness of the trained CNN and validate the coupling between visual clover content in the canopy and clover dry matter fraction, the best-performing FCN-8s CNN was utilized to semantically segment the images from the 179 sample pairs.

**Figure 9:**
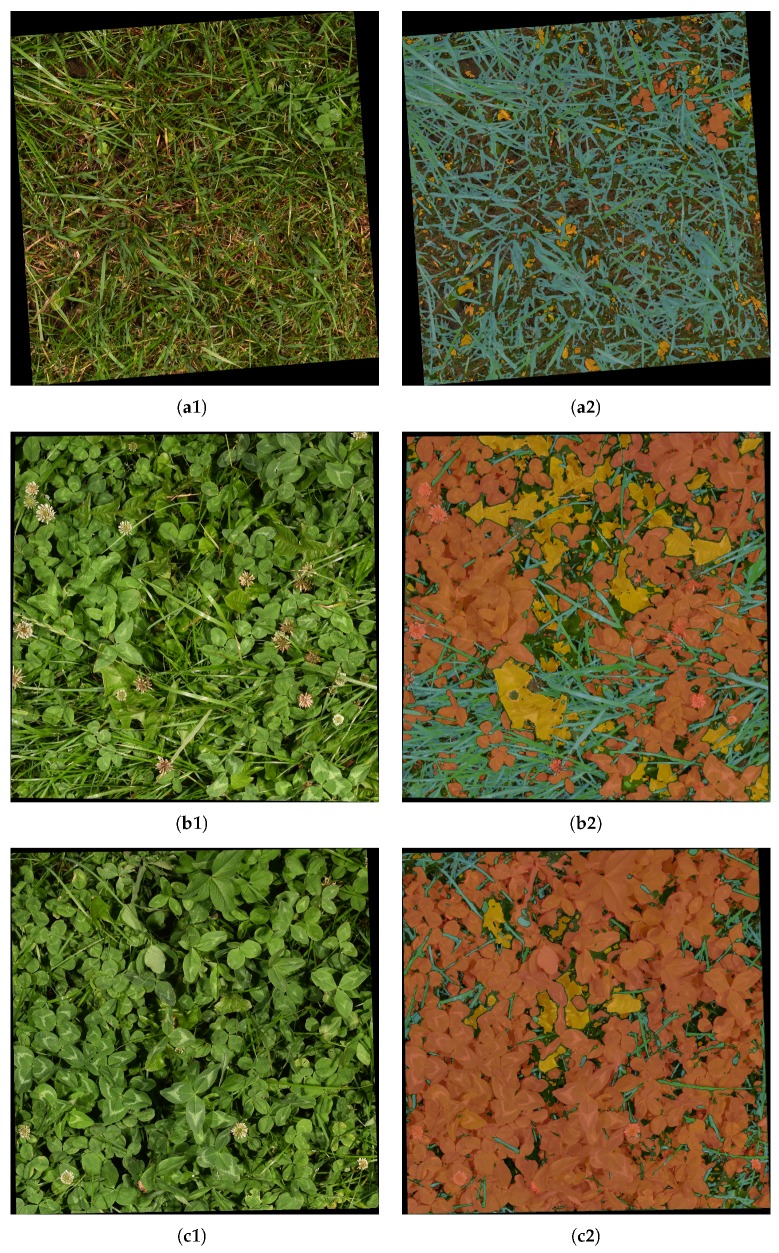
Visualization of CNN-driven semantic segmentation of highlighted sample pairs from Figure 10. The first column shows the evaluated image. The second column shows the thresholded identification of grass (blue), clover (red), and weeds (yellow) in the image. (**a1**) Low clover fraction; (**a2**) Semantic segmentation of (a1); (**b1**) Medium clover fraction; (**b2**) Semantic segmentation of (b1); (**c1**) High clover fraction; (**c2**) Semantic segmentation of (c1).

In contrast to the isolated semantic segmentation case of Section 3.1, it was beneficial to increase the stability of the clover-grass analysis at the cost of sparsity in the semantic segmentation. It was better for shadow regions and foreign objects to be ignored than falsely classified, as they influenced the dry matter composition estimation. To apply this, the traditional non-max suppression used for semantic segmentation was substituted by a custom threshold on the individual softmax score maps of the CNN. By qualitative visual inspection of five images, the softmax thresholds were defined as 0.95, 0.8, and 0.3 for clover, weeds, and grass, respectively. This corresponds well to the overestimation of clover and underestimation of grass in Figure 8 in Section 3.1. Three samples of the thresholded segmentation of clover-grass images are shown in Figure 9.

Qualitatively, the three samples of semantic segmentation demonstrated an excellent understanding of the images by the convolutional neural network across vegetation densities and clover fractions. Visible weeds, clover, and grasses were detected with high accuracy while withered vegetation and ground were correctly ignored.

The coupling between the clover-grass images and dry matter composition was evaluated using the clover fraction metrics defined in Equations (1) and (2) for dry matter samples and image samples, respectively:

(1) $$fraction_{clover,\;DM}=\frac{clover_{dry\;matter}}{vegetation_{dry\;matter}}$$

(2) $$fraction_{clover,\;px}=\frac{N_{clover,\;pixel}}{N_{vegetation,\;pixel}}$$

**Figure 10:**
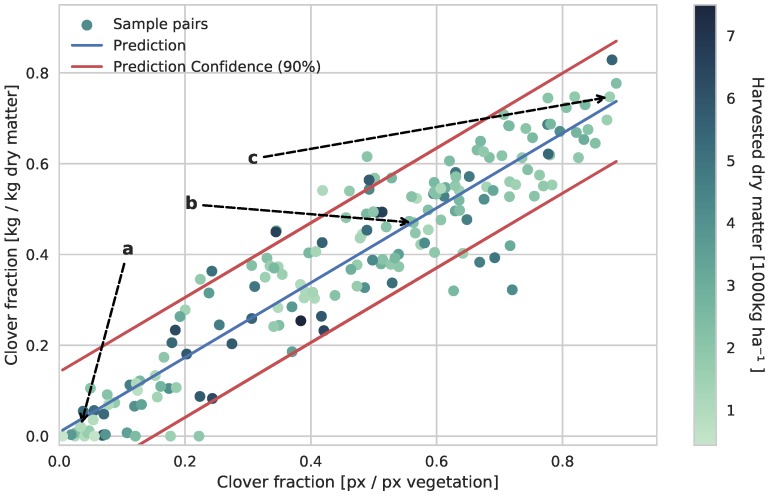
Comparison and linear regression of the visual clover ratio determined by the CNN and the actual clover ratio in the harvested dry matter at the primary field site. The linear relationship between the visual and dry matter fraction of clover in the sample pairs is clear. The annotations *a*, *b*, and *c* refer to the three image samples shown in Figure 9.

The result of the automated CNN-driven image analysis of visual clover content is shown in Figure 10. The relationship between the CNN-driven analysis and the dry matter clover ratio in the photographed clover-grass patches was eminent and similar to the manually obtained relationship shown by Himstedt et al. [14]. The linear regression based on the 179 primary samples pairs led to a clover dry matter fraction prediction with a standard deviation of 7.8%. This was achieved while covering the spread of total yield, clover fraction, weed levels, fraction of red and white clover, and time in season of the sample pairs.

Besides the general fitness of the linear regression, multiple outliers appeared in Figure 10. Looking into the underlying image samples and image analysis, two distinct causes were observed.

1. The image was out of focus in sparse or low-yield vegetation.
  The low-yield outliers along the x-axis all suffered from overestimated clover content. Due to the loss of focus in these samples, blurred vegetation on the ground was often misclassified as clover.
2. The clover-covered area of the canopy did not always represent the dry matter composition. In the range of 30–60% dry matter clover fraction, the canopy could be less representative of the harvested samples. This was the result of the heavy occlusions and was directly linked to the challenges of using the top-down view. The furthest outlier (0.75,0.32) was for example a dense clover grass mixture, covered by a shallow layer of clover leaves, giving the impression of a much higher clover fraction.

**Figure 11:**
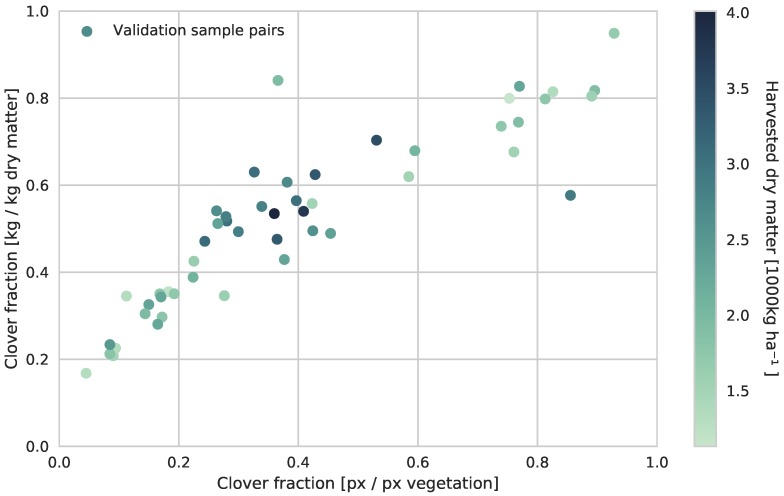
Comparison of the visual clover ratio and the dry matter clover ratio of the validation field site. The linear relationship is more poorly defined, with an introduction of a noticeable offset, possibly due to a less complete segmentation of clovers in the image samples.

The trained CNN was also evaluated on the validation field located 210 km from the primary field site, while preserving the threshold parameters. The sample pair relationship of the validation field is shown in Figure 11. When comparing the results of the two field sites, the validation site led to a similar relationship, with an introduction of an offset. This offset shifts the validation sample pairs towards the upper region of the prediction confidence of the estimator in Figure 10, leading to a general underestimation of the clover fraction in the validation field.

## 4. Discussion

The ability to train a CNN for image segmentation using solely simulated images has been shown to have good prospects, as it allows one to train a network for tasks for which it would otherwise be unfeasible to achieve the needed amount of data. The acquired 195 plant samples for training have been additionally been reduced by 75%, leading to a pixelwise accuracy drop of 1.2 percentage points. This drop is mainly a result of worsened distinction between clover and weeds, caused by the use of only six weeds. This low number of samples does not cover the variation of weeds in the test data in terms of number of neither species nor appearances. This demonstrates that high classification accuracies can be achieved on real images with only few plant samples used for training, and this translates to reaching a working prototype for semantic segmentation within hours with manual labor, as opposed to weeks or months if following a traditional work flow of manual image labeling.

Care should be taken when training the convolutional neural network to span the variations in the test data by training using the corresponding variations in the training data. This was the case for vegetation density and varying ratios of clover, grass, and weeds in the simulated images. More care should be taken when simulating training data to imitate natural and common errors, introduced when collecting images. These include lighting conditions, color temperature, image noise, and blurring. Several of the images with a high ratio of misclassified pixels were blurred. As the network was trained on sharp images, it is believed that the number of misclassifications can be reduced by introducing blurred simulated images in the training data.

From the estimate of the clover content of the dry matter we see that the uncertainty is larger in cases where the clover and grass are mixed compared to cases dominated by one species. This is because the camera can only see the canopy of the plant cover, and plants of one species that is hidden by the others would thereby make the estimated ratio less accurate. In cases where the sward is dominated by one species, the estimated dry matter-ratio would be less affected by this phenomenon. Nonetheless, the estimated ratio is close to the real ratio even in cases where the real ratio is close to 50%.

When evaluating the system on the validation field at a separate location, the automated image-based estimation of the dry matter clover fraction does not translate accurately between the two field sites. The design of the two field trials differs largely, mainly in terms of absence of red clover in the validation field seed mixture, fertilization strategies, organic or conventional farming, and variation of cultivars in the plots. Through experiments outside of this paper, it has been shown that the system accurately translates to the validation field when lowering the threshold value for detecting clover pixels from 0.95 to 0.85. This suggests a visual difference between the clovers in the two field sites, leading to partly unclassified clovers. By introducing larger variations of clovers from multiple locations in the image simulation, the CNN should be generalized to better handle natural visual variances between the fields.

It is essential to be aware of the accuracy of alternative methods for estimation the botanical dry matter composition. While separating a forage sample into species by hand does provide the ground truth composition for this paper, this is not feasible for real applications. Fair comparisons include vision-based evaluation by expert consultants in the field and near-infrared reflectance spectroscopy (NIRS)-based estimation, often integrated in grass sward harvesters used for research.

While the visual estimation accuracy of consultants remains undocumented, this method is time consuming and requires the consultant to inspect the clover-grass throughout the field and map it accordingly. NIRS-based methods, such as in [23], show comparable estimation accuracy of grass swards of either white clover or red clover, by use of distinct models for each case. The accuracy on grassland swards with mixed red and white clover species, as in this experiment, has not been investigated.

To utilize the botanical composition of clover-grass leys for targeted fertilization, the information must be available at the time of fertilization. The largest amount of fertilizer is typically applied in the spring prior to the start of the growth period. At this stage NIRS for identifying the botanical composition is ruled out, since the crop is too small for harvesting. This leaves a great potential for the non-destructive approach of monitoring clover-grass mixtures in this paper. Future work includes extending the presented method for clover content estimation to preseason image samples. This extension allows the camera-based system to provide the necessary botanical information for optimizing the fertilizing strategy, for every fertilization. Following the approach presented in this paper, the image analysis of the extension can be operational with a couple of days of labor for gathering relevant image samples and cropping out representative plant samples for data simulation. Other future work relates to extending the parametric information level delivered by the convolutional neural network to further improve the dry matter composition estimates, or directly predicting the dry matter botanical composition in the neural network.

## 5. Conclusions

It has been shown that a fully convolutional neural network can be trained to semantically segment images of clover-grass swards using only simulated images. This fully convolutional neural network has been proven capable of discriminating between clover, grass, and weeds in real images at a pixel level with high accuracies.

The clover fraction that is estimated from the segmentation has been shown to be directly coupled to the clover fraction of the dry matter, making the method a cheap, nondestructive, and applicable method for determining the clover fraction, which is needed to fertilize optimally. Furthermore, it presents a research tool for investigating clover and grass dynamics in mixed swards more efficiently and with better spatial and temporal resolution than is possible with laborious separation by hand.

## Figures and Tables

**Table 1:** Comparison of the approach by Bonesmo et al. [11] and the obtained results of FCN-8s on simulated images. Our results are presented when using all 195 plant samples (100%) to simulate clover grass images and when using only 25% of the samples. IoU: intersection over union. F.w: frequency weighted.

Method	Trained Plant Samples	Pixel Accuracy	Mean IoU	F.w. IoU
Bonesmo et al. [11]	-	65.5	34.3	47.9
FCN-8s	25%	82.2	62.4	70.7
FCN-8s	100%	83.4	65.5	71.7

## Supplementary Materials

The following are available online at www.mdpi.com/1424-8220/17/12/2930/s1, Figure 1: Example of the image analysis on a real image. Each element in the image is analyzed and classified as either grass (blue), clover (red), weeds (yellow) or unidentified (black overlay). (a) Example input image. (b) Automatically analyzed image.

## Abbreviations

The following abbreviations are used in this manuscript:

CNN	Convolutional neural network
FCN-8	Fully convolutional network with a output stride of 8. [17]
HSL	Hue, saturation, and lightness
IoU	Intersection over union. Also known as the Jaccard index.
LED	Light-emitting diode
N	Nitrogen
NIRS	Near-infrared reflectance spectroscopy
RGB	Red, green, and blue
WGS84	World geodetic system 1984
