# Supplementary material for: Estimation of the Botanical Composition of Clover-Grass Leys from RGB Images Using Data Simulation and Fully Convolutional Neural Networks
Source: Sensors (Basel). 2017 Dec 17;17(12):2930. doi: 10.3390/s17122930 (PMC5751073; doi:10.3390/s17122930)
Supplement: Supplementary file 1 [file sensors-17-02930-s001.pdf]

Supplementary Materials: Estimation of the Botanical Composition of Clover-Grass Leys from RGB Images Using Data Simulation and Fully Convolutional Neural Networks

Søren Skovsen [1,*], Mads Dyrmann [1], Anders Krogh Mortensen [2], Kim Arild Steen [3], Ole Green [3], Jørgen Eriksen [4], René Gislum [2], Rasmus Nyholm Jørgensen [1] and Henrik Karstoft [1]

1 Department of Engineering, Aarhus University, Finlandsgade 22, 8200 Aarhus N, Denmark; madsdyrmann@eng.au.dk (M.D.); rnj@eng.au.dk (R.N.J.); hka@eng.au.dk (H.K.)
2 Department of Agroecology, Aarhus University, Forsøgsvej 1, 4200 Slagelse, Denmark; anmo@agro.au.dk (A.K.M.); rg@agro.au.dk (R.G.)
3 Agro Intelligence ApS, Agro Food Park 13, 8200 Aarhus N, Denmark; kas@agrointelli.com (K.A.S.); olg@agrointelli.com (O.G.)
4 Department of Agroecology, Aarhus University, Blichers Allé 20, 8830 Tjele, Denmark; jorgen.eriksen@agro.au.dk
* Correspondence: ssk@eng.au.dk
Received: 31 October 2017; Accepted: 12 December 2017; Published: 17 December 2017

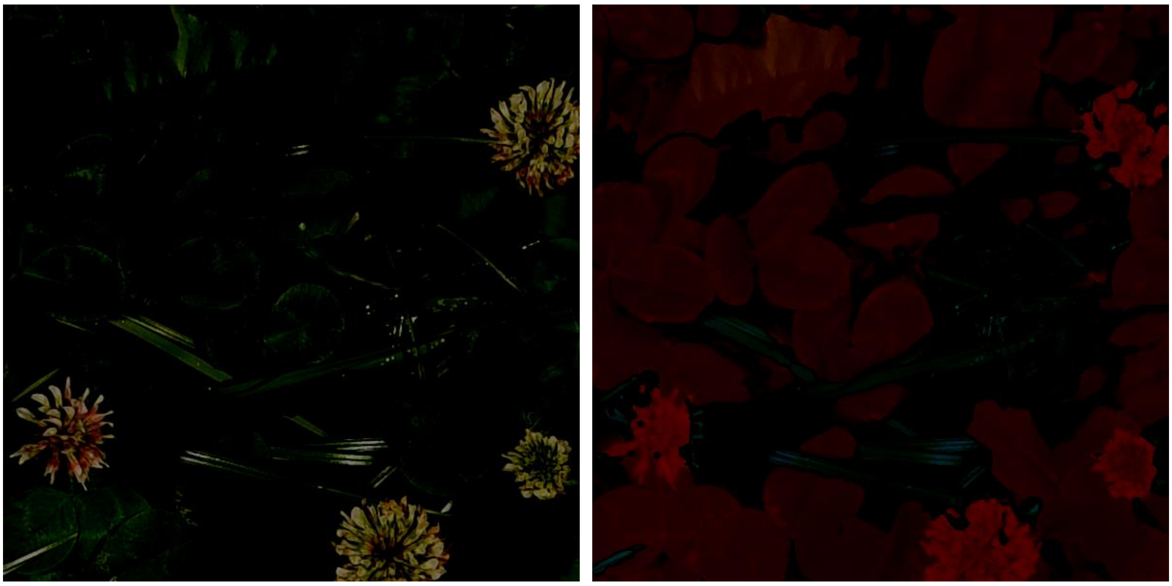

(a) (b)

Figure 1. Example of the image analysis on a real image. Each element in the image is analyzed and classified as either grass (blue), clover (red), weeds (yellow) or unidentified (black overlay). (**a**) Example input image. (**b**) Automatically analyzed image.
